# Potential Effects of Remdesivir on Tacrolimus Exposure in Transplant Recipients With COVID-19 Infection

**DOI:** 10.1016/j.ekir.2023.04.028

**Published:** 2023-04-30

**Authors:** Ehsan Habeeb, Steven Gabardi, Keri Townsend, Miae Kim

**Affiliations:** 1 Brigham and Women’s Hospital, Boston, Massachusetts, USA; 2 Department of Clinical Pharmacy, College of Pharmacy, Taibah University, Madinah, Kingdom of Saudi Arabia; 3 Harvard Medical School, Boston, Massachusetts, USA

**Keywords:** calcineurin inhibitor, COVID-19 infection, cyclosporin, interaction, remdesivir, tacrolimus

## Abstract

**Introduction:**

Remdesivir has proven to have benefits against COVID-19 infection. However, data supporting drug-drug interactions are insufficient. Clinicians have noticed that calcineurin inhibitor (CNI) levels tend to change after starting remdesivir. This retrospective study aimed to evaluate the effect of remdesivir on CNI levels.

**Methods:**

This study included adult solid organ transplant recipients hospitalized for COVID-19 who received remdesivir while on CNI. Patients were excluded if they started on other medications known to interact with CNI. The primary end point was the percentage of change in CNI levels after starting remdesivir. Secondary end points included the time until CNI levels reached a maximum increase in trough levels, the incidence of acute kidney injury (AKI), and the time until CNI levels normalized.

**Results:**

Of the 86 patients screened, 61 were included (56 on tacrolimus and 5 on cyclosporine). Most patients received kidney transplants (44.3%), and baseline demographics were similar among the transplanted organs. The median increase in tacrolimus level after starting remdesivir was 84.8%, and only 3 patients had no significant change in CNI levels. The median increase in tacrolimus level was more pronounced in lung and kidney recipients than in heart recipients (96.5% vs. 93.9% vs. 64.6 %, respectively). The median time to maximum increase in tacrolimus trough levels was 3 days, and it took 10 days after the remdesivir course for levels to return to baseline.

**Conclusion:**

This retrospective analysis demonstrates that CNI levels were significantly elevated after starting remdesivir. However, future studies are warranted to evaluate this interaction further.

## Graphical abstract

**Figure:**
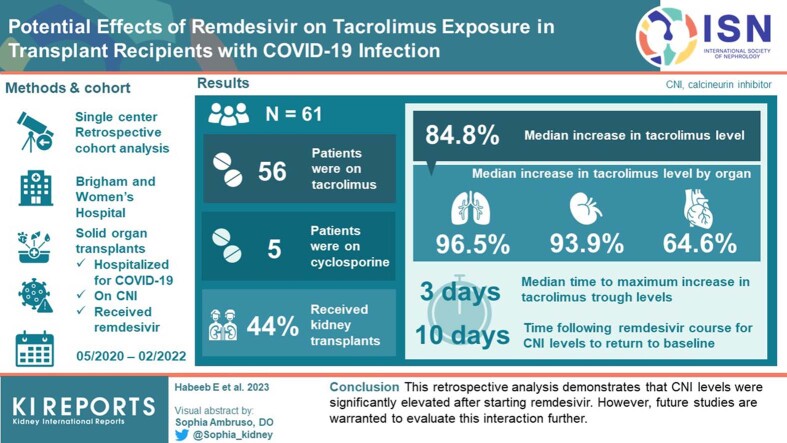

Transplant recipients have a markedly increased risk of infection because of prolonged use of immunosuppressants.^1^ Furthermore, they seem to be at a particularly high risk for progressing to severe COVID-19 infection, given their comorbid conditions and frequent contact with health care facilities.^2^ Antiviral and anti-inflammatory agents have been the most validated therapeutics for patients hospitalized with COVID-19 infection.^3^^,^^4^ Among these various agents, remdesivir has become a prominent therapy for hospitalized patients. It is a broad-spectrum antiviral that interferes with the genome of the Coronavirus and prevents the virus from replication. Remdesivir acts as a nucleoside analog and inhibits RNA-dependent RNA polymerase of the virus.^3^ When compared to placebo, many clinical trials have shown that remdesivir can shorten the time to recovery in patients hospitalized with COVID-19, especially in patients with a high likelihood of disease progression.^5^^,^^6^ Therefore, remdesivir has become a first-line treatment for early COVID-19 infection among this patient population. Despite this, there are many concerns with the drug-drug interaction (DDI) potential of remdesivir, because it could interact with multiple medications via cytochrome P450 3A4 and permeability glycoprotein (P-gp) inhibition.^7^^,^^8^

CNIs are mainstays of maintenance immunosuppressants in solid organ transplant recipients.^9^ Following oral administration, CNI are extensively metabolized by CYP3A5 and CYP3A4 in gut and liver, and they are also substrates of P-gp, conferring a high potential for DDI.^10^ The consequence of inhibiting these metabolizing enzymes by remdesivir may be an increase in the risk of over-immunosuppression by CNI. Alongside the narrow therapeutic index of CNI, this interaction can further potentiate the risk of infections and drug toxicities, such as AKI.^10^ Clinicians have noticed the trend of increased CNI exposure when patients were initiated on remdesivir. However, there is a lack of data evaluating this possible DDI.

It is of utmost importance to assess the extent and timing of the potential DDI between remdesivir and CNI to guide clinicians to safely use remdesivir in solid transplant recipients. This study aimed to assess the impact and duration of this DDI.

## Methods

### Study Design

This study was a single-center, retrospective cohort analysis conducted at Brigham and Women’s Hospital, a tertiary care academic medical center. Patients were enrolled from May 2020 to February 2022 using system-generated reports from the electronic health record. Approval from the Mass General Brigham Institutional Review Board was obtained before initiating the study.

Patients were included if they were 18 years of age or older with a history of solid organ transplant and received both remdesivir and a CNI (tacrolimus or cyclosporine). Patients were excluded if they started or discontinued any additional medications known to interact with CNI (for example, letermovir, ritonavir, any of the azole antifungals, rifampin, erythromycin, clarithromycin, phenytoin, diltiazem, and verapamil). Local institutional guidelines recommend different courses of remdesivir on the basis of the severity of the infection. The usual course can last from 3 to 10 days of 100 mg daily of remdesivir, including an initial loading dose of 200 mg. Baseline CNI levels before admission were used as a control group because there were only 3 patients who were admitted with COVID-19 infection and did not receive remdesivir during the admission, which could not be used as a control group because they were admitted only for 1 or 2 days. Baseline levels of CNI were identified as the average of the last 3 levels in the past 6 months before starting remdesivir. Trough levels were identified and accounted for if they were collected within 2 hours of the proper trough timing (i.e., 22–26 hours with daily CNI and 10–14 hours with twice daily CNI). During the admission, CNI trough levels were mainly checked daily. The maximum increase in CNI trough levels is referred to as the peak. The peak is identified as the highest level before making any changes to the home dose. Dose-normalized trough concentration (trough divided by the dose) was investigated to assess the effect size of DDI. It was also used to calculate the duration of the DDI. The duration of DDI was defined as the time (in days) from the change of the baseline dose-normalized trough concentration after starting remdesivir until the time for the dose-normalized trough concentration to return to baseline. We considered the patient to be back to baseline if the dose-normalized trough concentration was within 20% of that at the baseline.

### Study Outcomes

The primary end point was to evaluate the percentage change in CNI levels after starting remdesivir. The evaluated secondary end points were time to maximum increase in tacrolimus trough levels, the number of patients who required dose modification, the incidence of diarrhea as a presenting symptom, the correlation of tacrolimus trough concentration, incidence of AKI (on the basis of the definition of Kidney Disease Improving Global Outcomes of an increase of >0.3 mg/dl in serum creatinine within 48 hours, or an increase in serum creatinine of >1.5 mg/dl times baseline within 7 days), and the duration of DDI (examined by comparing dose-normalized trough concentrations before and after remdesivir). Furthermore, an analysis was performed to assess the concomitant use of steroids (6 mg of dexamethasone or an equivalent steroid) while on the remdesivir course to evaluate if they further potentiate the DDI. In addition, another analysis for the primary end point was performed comparing patients with diarrhea versus those without diarrhea as a presenting symptom. This subgroup analysis was also performed to determine differences between the transplanted organs.

### Data Collection

Data were collected by chart review. Baseline data included age, gender, body mass index, duration of hospital stay, interacting medications, the number of patients admitted to an intensive care unit, diarrhea and oxygen support at presentation, transplant date, and transplanted organ. Furthermore, daily trough levels, the dose of CNI, duration of remdesivir course, the total milligram of remdesivir during the admission, on steroids while on remdesivir, and other laboratory parameters, including liver function tests, inflammatory markers, and serum creatinine before admission and daily during admission.

### Statistical Analysis

Median with interquartile range was used in reporting continuous data given the skewed distribution for the variables. Mode with percentile was used in reporting nominal data. Two-sided Mann-Whitney *U* or Wilcoxon signed-rank tests were used as appropriate for continuous data. χ^2^ or McNemar’s tests were used as appropriate in analyzing the reported nominal data. All *P*-values were 2-sided, and *P* ≤ 0.05 was considered statistically significant.

## Results

### Included Patients

**Figure 1:**
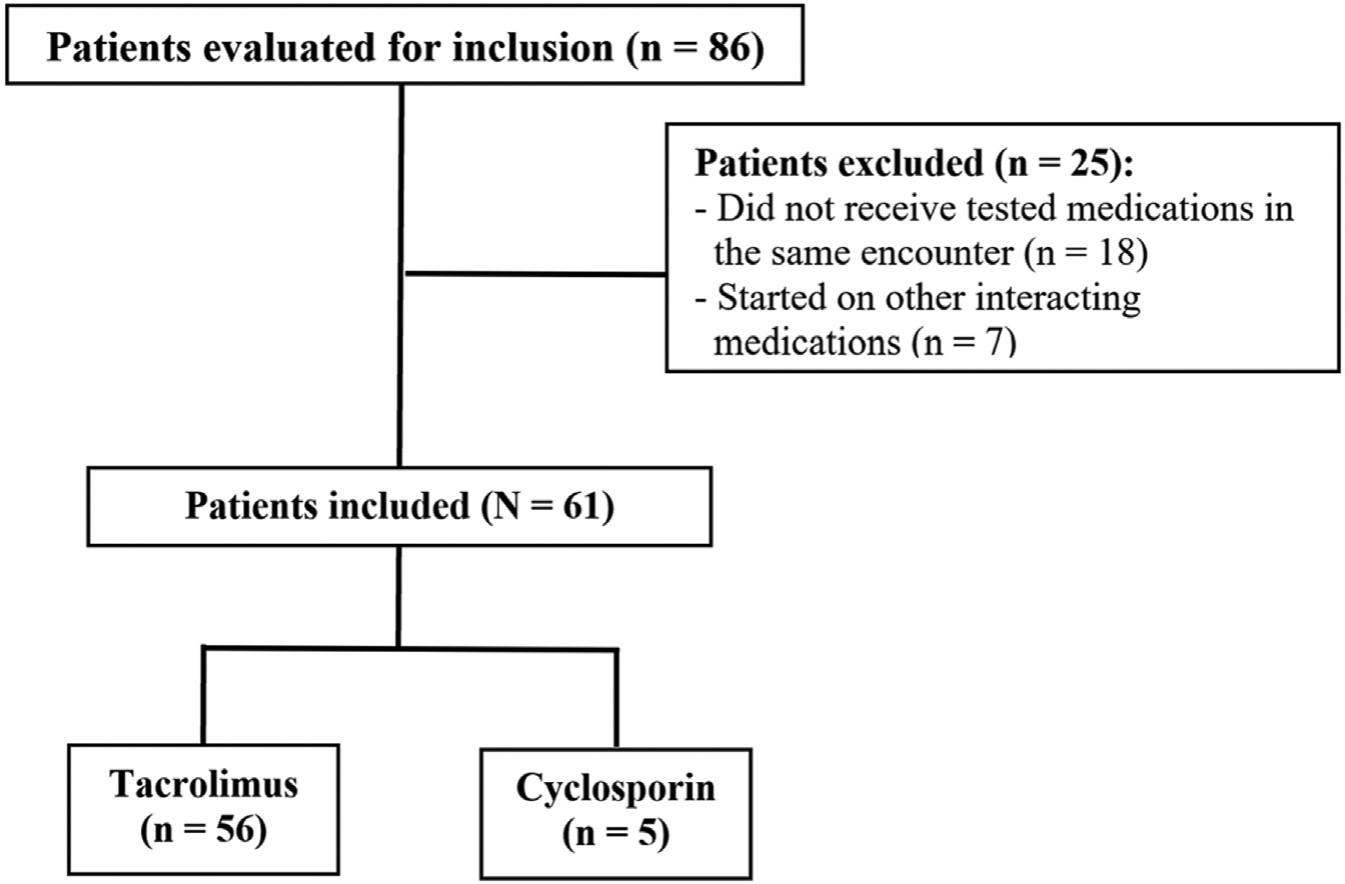
Patient enrolment.

Eighty-nine solid organ transplant recipients were admitted with a diagnosis of COVID-19 during the study period. Of these, 3 patients had not received remdesivir during admission, and 86 were screened for inclusion. Of those, 18 patients were excluded because they did not receive CNI during admission, and 7 patients were excluded because they were started on medications that are known to interact with CNI during admission. Of the 61 patients included in the final analysis, 56 were on tacrolimus and 5 were on cyclosporine (91.8% vs. 8.2%, respectively, Figure 1). Most patients completed a 5-day course of remdesivir (77.1%), and all included patients received a loading dose of 200 mg of remdesivir (Table 1). In addition, 70.5% of the patients received steroids while on the remdesivir course. Only 3 patients were admitted to an intensive care unit (4.9%). All patients were more than 2 years out from their transplant.

**Table 1:** Baseline characteristics

Variables	Included patients (*N* = 61)
Age (yr)^a^	60 [49:72]
Gender, male^b^	39 (63.9)
BMI (kg/m^2^)^a^	27.2 [23:32.6]
Transplanted Organ^b^	
Kidney	27 (44.3)
Heart	20 (32.8)
Lung	14 (22.9)
Years since transplant^b^	
<5 yr	25 (41)
>5 yr	36 (59)
Remdesivir course of treatment, d^b^	
3 d	8 (13.1)
5 d	47 (77.1)
10 d	6 (9.8)
On steroids while on remdesivir^b^	43 (70.5)
ICU admission^b^	3 (4.9)
Oxygen support at presentation^b^	
Room air	38 (62.3)
Nasal canula	21 (34.4)
Mechanical ventilation	2 (3.3)
Diarrhea at presentation^b^	7 (11.5)
Laboratory parameters at presentation^a^	
AST, IU/l	28 [18:41]
ALT, IU/l	17 [11:34]
CRP, mg/l	76 [44:127]
ESR, mm/h	54 [33:76]
Baseline use of HD^b^	6 (9.8)
Serum creatinine, mg/dl	(*n* = 55)
Baseline	1.3 [0.9:1.7]
At presentation	1.57 [1.19:2.5]
Highest CNI level	1.71 [1.29:2.9]

BMI, body mass index; ICU, intensive care unit; AST, aspartate aminotransferase; ALT, alanine transaminase; CRP, C-reactive protein; ESR, erythrocyte sedimentation rate; HD, hemodialysis.

Steroids = 6 mg of dexamethasone or equivalent.

a Median [interquartile range].

b n (%).

Baseline characteristics for the final analysis include (Table 1) the following: a median age of 60 years with 64% male and a median body mass index of 27.2 kg/m^2^. Most included patients were kidney recipients, followed by heart recipients, then lung recipients (44.3% vs. 32.8% vs. 22.9%, respectively). All other baseline characteristics are described in Table 1.

### Outcomes

As shown in Table 2 for the primary outcome, the median for peak tacrolimus troughs level almost doubled after starting remdesivir when compared to the baseline levels (10.9 ng/ml [8.8–14] vs. 6 ng/ml [4.5–7], *P* < 0.05), with a median percent increase of 84.8% (range 61%–232.3%). The median cyclosporine trough level was also increased but to a less significant extent (179 ng/ml [89–198] vs. 112 ng/ml [91.5–157.5]), with a median percent increase of 19%. Most patients (69%) had their peak levels at day 3 of remdesivir, and few patients had their peak after day 3 (8.1%) as shown in Table 2. There were 7 patients who had diarrhea at admission; all of them were receiving tacrolimus. On further analysis for the primary end point comparing CNI levels for those presenting with diarrhea to those without diarrhea, peak CNI levels were higher in patients without diarrhea (9.6 ng/ml [8.9–11.7] vs. 11.2 ng/ml [8.2–14.8]). Considering that there were only 5 patients who received cyclosporine in the study, those patients were not included in the secondary analyses. Interestingly, no changes in the mean CNI levels were noted for the 3 patients who did not receive remdesivir during their short admission (baseline vs. peak, 5.8 ng/ml ± 1.2 vs. 6.1 ng/ml ± 0.4).

**Table 2:** Primary end point

CNI trough levels	Baseline levels	Peak levels after starting remdesivir	Percentage of increase	*P*-value
Tacrolimus levels (*n* = 56)^a^	6 ng/ml [4.5–7]	10.9 ng/ml [8.9–14]	84.8% [61–232.3]	0.0001
Cyclosporin levels (*n* = 5)^a^	112 ng/ml [91.5–157.5]	179 ng/ml [89–198]	19% [8.6–49]	-
Day when peak levels were reached^b^	Included patients (61)			
Day 1 on remdesivir	1 (1.7)			
Day 2 on remdesivir	10 (16.4)			
Day 3 on remdesivir	42 (68.9)			
Day 4 on remdesivir	3 (4.9)			
Day 5 on remdesivir	2 (3.2)			
No changes on CNI levels while on remdesivir	3 (4.9)			

CNI, calcineurin inhibitors.

a Median [interquartile range].

b n (%).

**Figure 2:**
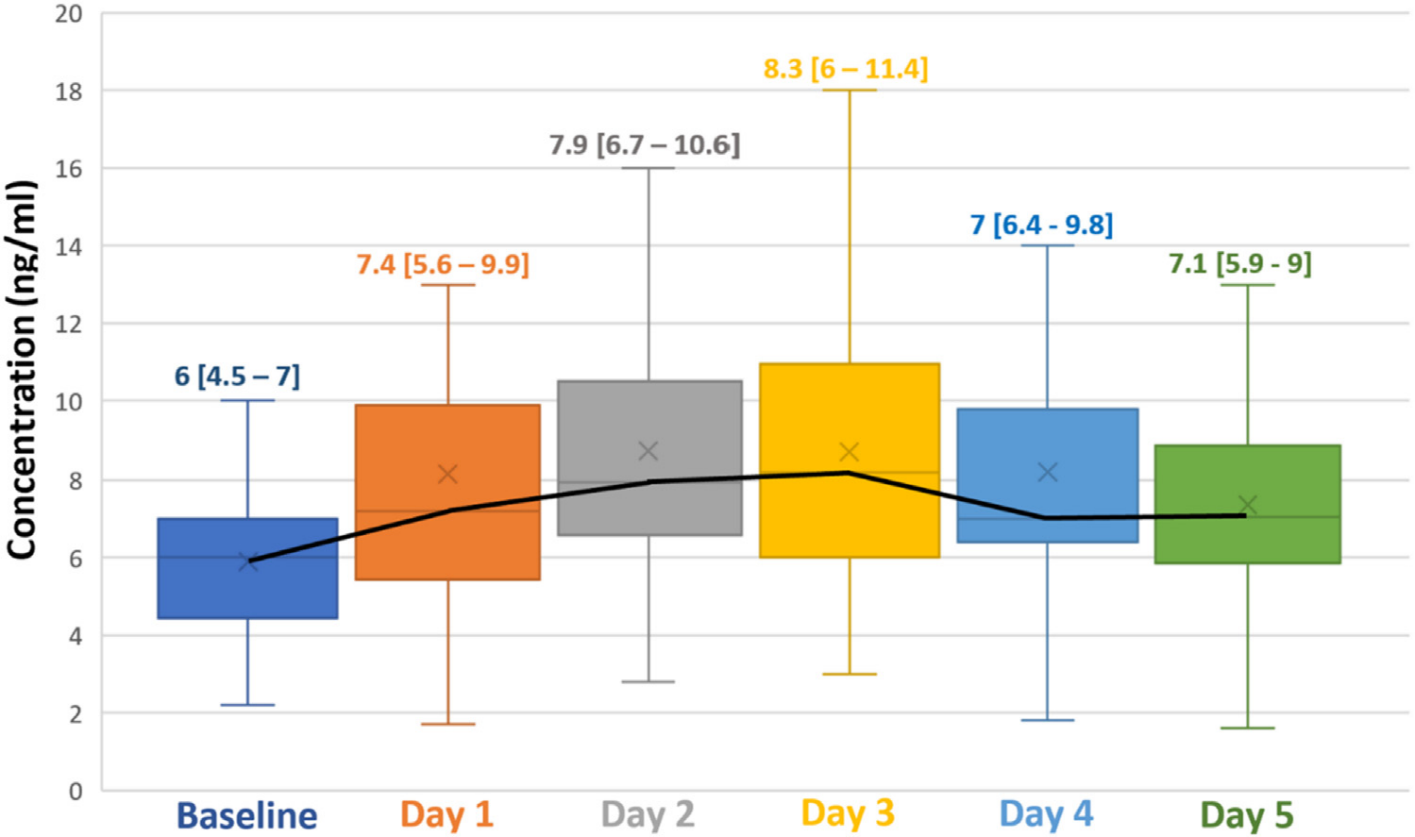
Tacrolimus troughs during admission: box and whisker plot represents the median with interquartile range. The X in the middle of the box represents the mean. The black line represents the trend of the daily median.

As shown in Figure 2, the median level for tacrolimus trough at baseline was 6 ng/ml. The median trough levels continued to increase from baseline after starting remdesivir until day 3 of treatment (6 ng/ml [4.5:7] vs. 8.3 ng/ml [6:11.4], *P* < 0.05). On day 3, about 70% of the patients had a reduction in the home dose, or CNI was held. The median time (in days) when CNI reached a maximum increase in trough levels after starting remdesivir was 3 days (1.5:5), suggesting that this DDI peak occurred quickly. All included patients had no changes in home doses before day 3 of admission.

**Figure 3:**
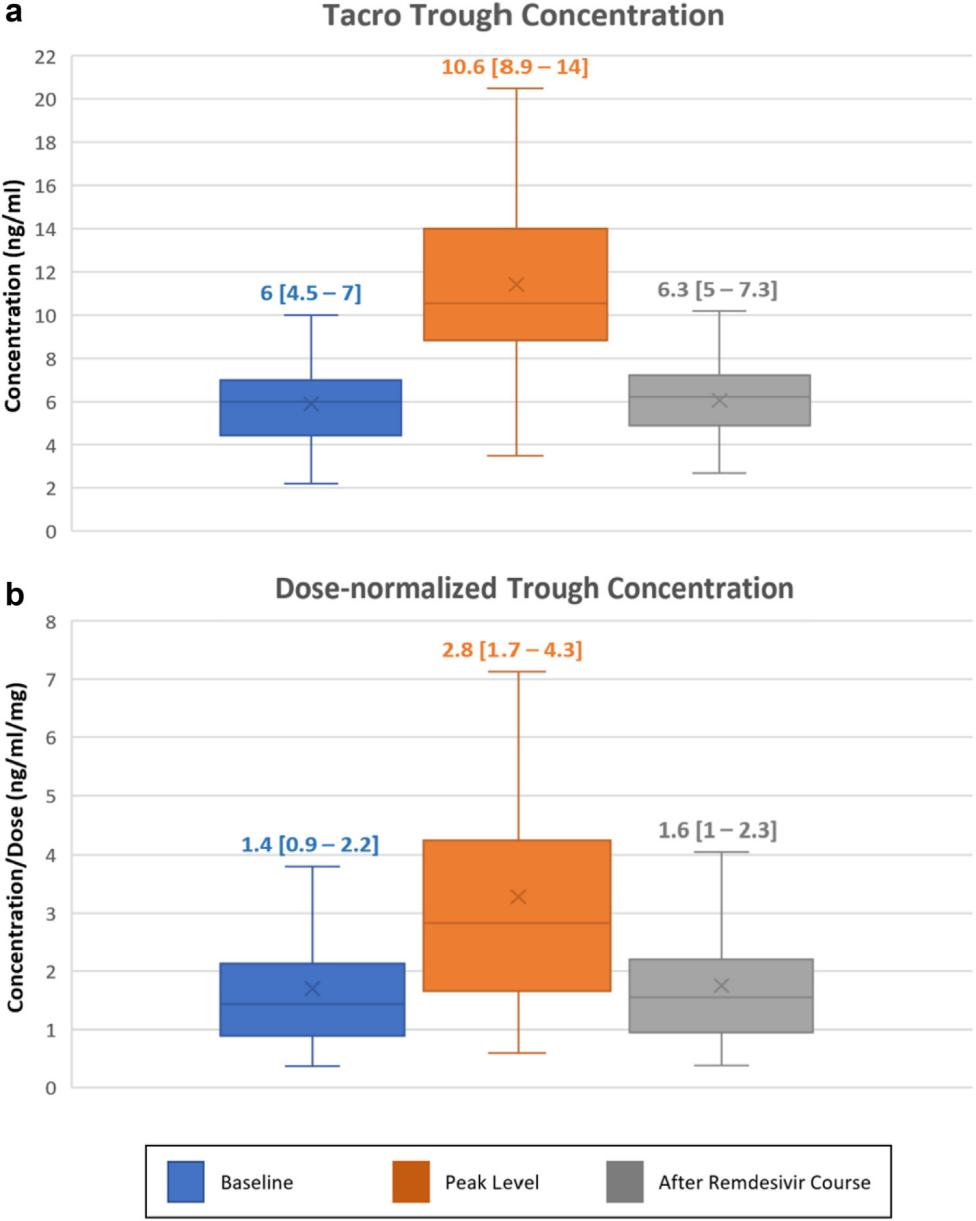
Changes in trough concentration and dose-normalized trough concentration: box and whisker plot represent the median with interquartile range, blue color represents baseline levels, orange color represents peak levels, and gray color represents levels after the remdesivir course

A comparison of tacrolimus trough levels and dose-normalized trough concentration at baseline, peak levels before any changes in home doses, and after completing the remdesivir course is shown in Figure 3. The median tacrolimus trough concentration significantly increased compared to the baseline before starting the remdesivir course (6 ng/ml [4.5:7] vs. 10.6 [8.9:14,] *P* < 0.05). The median dose-normalized trough concentrations increased by double the baseline after starting the remdesivir course (1.4 ng/ml/mg [0.9:2.2] vs. 2.8 ng/ml/mg [1.7:4.2], *P* < 0.05). There were frequent dose interruptions because of the great extent of the observed DDI. The peak dose-normalized trough concentrations were taken before any dose adjustment to reflect the effect of the dose on the troughs correctly. The median time for dose-normalized trough concentration to return to baseline was 10 days [5:16] after completing the remdesivir course. Most patients on tacrolimus had an increase in trough concentration ranging from 51% to 100% (44.6%), followed by patients who had an increase of 101% to 150% (25%). Patients who had a minor or very major change in tacrolimus trough were less frequent (12.5%) with an increase in trough concentration of <50% and >200%. However, 5.4% of patients had no changes in tacrolimus trough level. Therefore, tacrolimus levels increased after starting remdesivir for all patients except for 3. These 3 patients were on a 3-day course of remdesivir. Other secondary end points are shown in Table 3.

**Table 3:** Secondary end points for patients on tacrolimus

Variables	Included patients (*N* = 56)
Number of days until CNI reached max trough levels^a^	3 [1.5:5]
Percentage of increase per organ received (%)^a^	
Kidney (*n* = 25)	93.9 [72.4–138.1]
Heart (*n* = 18)	64.6 [58.7–91.7]
Lung (*n* = 13)	96.5 [64.9–166.4]
Number of patients needed to reduce the dose^b^	32 (57)
Number of patients needed to hold the dose^b^	7 (12.5)
Incidence of AKI (%)^b^	44 (78.6)
Kidney (*n* = 25)	21 (84)
Heart (*n* = 18)	13 (72.2)
Lung (*n* = 13)	10 (76.9)
Number of days until CNI levels normalized^a^	10 [5:16]
On steroids while on remdesivir^b^	39 (69.6)

Percentage of increase and steroid use^a^	With steroid (*n* = 39)	Without steroids (*n* = 17)	*P-*value
	84.8 [58–138.1]	81.6 [62.6–123]	0.58

AKI, acute kidney injury.

Steroids = 6 mg of dexamethasone or equivalent;

a Median [interquartile range].

b *n* (%).

An evaluation of the percentage of change in tacrolimus levels by recipient organ was performed. Lung and kidney transplant recipients had a higher median increase in tacrolimus levels than heart transplant recipients (96.5% vs. 93.9% vs. 64.6%, respectively). Excluding the 6 patients on hemodialysis at baseline, 78.6% had AKI. Kidney transplant recipients were the majority to have AKI followed by lung transplant recipients, then heart transplant recipients (84% vs. 76.9% vs. 72.2%, respectively). We performed a subgroup analysis of the patients who had AKI and found that 88% had an increase in tacrolimus trough of 51% to 150%, with an average time of 2 days until the incidence of AKI. However, there was no correlation between the incidence of AKI and the percentage increase in tacrolimus levels (R^2^ <0.01). Finally, 70% of the patients were on a steroid while on the remdesivir course. Nevertheless, there was no difference between the median for the percentage increase in tacrolimus trough levels with or without steroids (84.8% vs. 81.6%, *P* = 0.58).

## Discussion

In this study, we aimed to evaluate the possible DDI between CNI and remdesivir. This study showed that receiving remdesivir with tacrolimus was associated with a significant increase in tacrolimus trough levels. However, the sample size was too small to assess the effect of DDI on cyclosporine. The increase in tacrolimus level was noted in all patients included except for 3. Interestingly, these 3 patients only received a 3-day course of remdesivir. There was a total of 5 patients on tacrolimus who received a 3-day course of remdesivir. Most of these patients were discharged home before checking their tacrolimus level on the third day; therefore, it is possible that we did not have enough data to observe the interaction in them adequately. Nevertheless, tacrolimus levels were doubled by day 2 for the other 2 patients who were on a 3-day course of remdesivir. About 12% of the included patients presented with diarrhea at admission. However, peak CNI levels were higher for those presenting without diarrhea. This makes it unlikely for diarrhea to explain the increased exposure of CNI levels. The effect size of this DDI varies, with tacrolimus levels increasing from 61% to 232.3% after starting remdesivir. However, a substantial number of patients (38%) had an increase in tacrolimus levels of more than double, suggesting a significant DDI between tacrolimus and remdesivir. The peak of the DDI occurred quickly at about 3 days after remdesivir initiation. The effect of interaction lasted for a while after course completion, taking about 10 days after the course of remdesivir for the dose and levels to normalize.

Because of the high variation in tacrolimus peak levels and more than two-thirds of the patients (72%) having an incident of AKI in this study, it is speculated that at least partially, increased exposure to tacrolimus because of the DDI has contributed to this complication. However, we found no clear association between the incidence of AKI and the percentage of increase in tacrolimus levels. The increase in tacrolimus levels was more significant in lung and kidney transplant patients compared to heart transplant patients. On further analysis, kidney and lung transplant patients were the majority to have AKI, which may decrease the elimination of remdesivir and its metabolites to some extent, resulting in a more increase in tacrolimus levels. However, this outcome cannot predict a significant clinical difference between these groups, given the small sample size and the difference in tacrolimus goal trough levels within a group. In addition, only 3 patients were admitted to an intensive care unit, which may mean the study population in this analysis does not include severely ill COVID-19 cases. Moreover, there was no difference in the percentage increase in tacrolimus trough levels with or without the use of steroids concomitantly with the course of remdesivir, suggesting that DDI could happen regardless of the use of steroids.

The literature evaluating this DDI is insignificant and dispersed. The possibility of this DDI was evaluated in Buxeda *et al.*,^2^ where they found a significant decrease in tacrolimus levels after starting remdesivir. However, the sample size was very small, and tacrolimus doses were significantly reduced in 11 of 22 patients at admission before starting remdesivir. Similarly, in Elec *et al.*,^11^ tacrolimus was withdrawn or adjusted to lower trough levels in all patients before starting antiretrovirals. Otherwise, the incidence of AKI in this study was more than previously reported in patients receiving remdesivir for COVID-19.^7^

The mechanism of this potential DDI between CNI and remdesivir can be proposed through different mechanisms. Previous studies evaluating remdesivir have found that remdesivir can inhibit some CYP450 enzymes, specifically CYP3A4.^12^ Although, the potential for DDI of remdesivir was thought to be limited because of the rapid clearance of the drug, the effects of its active and inactive metabolites on these enzymes were not evaluated.^13^ On the other hand, CYP enzyme expression and activity are thought to be affected by some pathologic conditions such as infection and inflammation. Some studies suggest that patients with COVID-19 infection tend to have increased cytokine levels. In addition, CYP enzymes can be suppressed by an infection-related cytokine increase and inflammation, which could increase exposure to CNI.^14^ Salerno *et al.*^15^ found that patients with COVID-19 had increased tacrolimus trough levels without receiving remdesivir, suggesting that it may be related to the COVID-19 infection. However, the patients in Salerno *et al.*^15^ were more critically ill, therefore, under a more significant effect of cytokines compared to the population in this analysis. In addition, about a third of the patients in Salerno *et al.*^15^ started on other interacting medications concurrently on admission (azithromycin, fluconazole, voriconazole, clotrimazole, posaconazole, isavuconazole, and erythromycin). Furthermore, about half of the patients had diarrhea, which can further increase the exposure to tacrolimus. On the other hand, diarrhea was not a common symptom in the patients of this analysis because only 7 patients presented with diarrhea, and their peak CNI levels were lower than those without diarrhea.

Because of the high risk of progressing to severe disease in the solid organ transplant population, almost all patients admitted with COVID-19 infection in our institution received remdesivir. Therefore, this limits our ability to ensure whether increased exposure to tacrolimus was solely because of the DDI with remdesivir. However, no changes in tacrolimus levels were noted for the 3 patients with COVID-19 infection who were not started on remdesivir in this analysis. In addition, the severity of COVID-19 infection in this analysis was mild to moderate, with most patients not requiring any oxygen support and only 3 patients needing a short intensive care unit stay, which makes it unlikely that hyperinflammatory status, such as COVID-19-related cytokine storm, has contributed to the increased exposure to CNI. Furthermore, it is known that the duration of COVID-19 infection is prolonged to several weeks in immunocompromised patients. However, the duration until tacrolimus dose-normalized levels returned to the baseline was about 10 days after the remdesivir course. Of note, our clinicians did not notice similar changes in CNI levels in patients who have mild COVID-19 infection and who did not require inpatient admission and the use of remdesivir.

There are several limitations to our study. Although this study was among a few patients to evaluate the potential DDI between CNI and remdesivir, it was conducted at a single academic medical center and was retrospective. The sample size was small, and we could not evaluate the potential DDI with cyclosporine. A different control group could not be used because only 3 patients were admitted and they did not receive remdesivir. In addition, the different courses of remdesivir and CNI dose interruption made it challenging to capture the full effect size of this potential DDI. Even though the severity of COVID-19 infection in this analysis was mild to moderate, there is still a possibility that the infection itself could have slightly contributed to the elevated CNI levels. Although CNI levels were checked daily in most patients during admission, some patients were discharged early and did not have as frequent monitoring thereafter. In addition, this analysis focused on the pharmacokinetic effects and has not evaluated the downstream effect of lowering CNI dosing and the associated risk of rejection. However, it is difficult to evaluate this potential DDI prospectively in the clinical practice. This analysis will potentiate confirmatory DDI studies between remdesivir and tacrolimus.

In conclusion, solid organ transplant patients receiving CNI with COVID-19 infection had a significant increase in tacrolimus levels after starting remdesivir. Most patients had an increase of more than double in tacrolimus trough levels within 3 days of starting remdesivir. However, the mechanism of this potential drug-drug interaction needs to be clarified, and if other factors, such as infection, may potentiate this interaction further. Future controlled prospective *in vivo* studies are warranted to evaluate this DDI further.

## Disclosure

All the authors declared no competing interests. This retrospective research does not include an active intervention. All patient identifiable data were removed on completion of the study. The study was approved by the Institutional Review Board at Mass General Brigham, protocol number 2022P000287.
